# Rates, Diagnoses, and Predictors of Unplanned 30-Day Readmissions of Critical Care Survivors Hospitalized for Lung Involvement in Systemic Lupus Erythematosus: An Analysis of National Representative US Readmissions Data

**DOI:** 10.7759/cureus.73099

**Published:** 2024-11-05

**Authors:** Adeniyi Fagbenro, Emmanuel S Amadi, Fidelis E Uwumiro, Stafford O Nwebonyi, Queeneth C Edwards, Madeleine O Okere, Sorrentina V Awala, Ifeoluwa Falade, Chukwuebuka A Ekpunobi, Chinemere E Ekezie, Emah E Uboh, Joycelyn Adjei-Mensah, Osasumwen Osemwota

**Affiliations:** 1 Internal Medicine, Bowen University College of Health Sciences, Iwo, NGA; 2 Internal Medicine, Hallel Hospital Port Harcourt, Port Harcourt, NGA; 3 Internal Medicine, Prime Healthcare-Southern Regional Georgia (SRGA), Riverdale, USA; 4 Internal Medicine, Nnamdi Azikiwe University Teaching Hospital, Nnewi, NGA; 5 Internal Medicine, Georgia Southern University, Statesboro, USA; 6 Internal Medicine, University of Port Harcourt Teaching Hospital, Port Harcourt, NGA; 7 Internal Medicine, Kursk State Medical University, Kursk, RUS; 8 General Practice, Mersey and West Lancashire Teaching Hospitals, Boston, GBR; 9 Internal Medicine, University of Nigerian Teaching Hospital, Ituku-Ozalla, Enugu, NGA; 10 Internal Medicine, Madonna University College of Medicine, Elele, NGA; 11 Internal Medicine, College of Medicine, University of Lagos, Lagos, NGA; 12 Internal Medicine, Achimota Hospital, Achimota, GHA; 13 Internal Medicine, Western Illinois University, Macomb, USA

**Keywords:** bronchoscopy, critical illness, extracorporeal membrane oxygenation, hospital readmission, lung diseases, lupus erythematosus, mechanical ventilation, tracheostomy

## Abstract

Introduction/objectives: Systemic lupus erythematosus (SLE) is a chronic autoimmune disease that frequently involves the lungs, contributing to significant morbidity in hospitalized patients. Critical care survivors with lung involvement in SLE are at particularly high risk for unplanned hospital readmissions, which can reflect the complexity of their disease, which often affects multiple organs and requires immunosuppressive therapy that increases infection risk. Severe pulmonary complications, critical illness sequelae, and challenges in medication adherence or follow-up care further contribute to their vulnerability. These factors result in frequent complications and flare-ups, making unplanned readmissions common in this population. This study assessed rates, most common reasons, and predictors of all-cause and SLE-related 30-day readmission among critically ill patients hospitalized for lung involvement in SLE.

Methods: We analyzed the 2021 National Readmissions Database. Critically ill non-elective adult hospitalizations for lung involvement in SLE were identified for analysis using a combination of the ICD-10 diagnostic code for SLE with lung involvement (M32.13) and presence of any procedure codes for mechanical ventilation, tracheostomy, extracorporeal membrane oxygenation, or bronchoscopy. Non-lung-related SLE admissions, non-SLE-related lung disorders, patients with concomitant COPD, history of COVID-19 or severe asthma, patients transferred in from other hospitals or admitted for <24 hours, and patients with a DNR order were excluded. We used χ2 tests to compare baseline characteristics between readmissions and index hospitalizations. Stata ranking commands were used to identify the most recurrent diagnoses associated with 30-day readmissions. We used multivariate Cox regression analysis to identify independent predictors of readmissions.

Results: Out of 3,472 index hospitalizations analyzed, 2,641 were discharged alive. Five hundred ninety-three (593; 22.5%) readmissions occurred within 30 days. Lung involvement in SLE was the most common reason for readmission (137; 23.1% of readmissions). Approximately 31.9% (189) of readmissions were due to other SLE-related complications. Readmissions were associated with higher inpatient mortality (18 (3.1%) versus 43 (1.6%); P=0.022), longer hospital stay (8 versus 5.2 days; P<0.001), younger mean age (26 versus 31 years; P=0.010), higher mean hospital costs (US $84,830 versus $64,628; P<0.001), and higher prevalence of heart failure (146 (24.6%) versus 526 (19.6%); P=0.024), CKD (435 (73.3%) versus 1,573 (58.6%); P<0.001), and anemia (138 (23.2%) versus 432 (16.1%); P=0.003) compared with index hospitalizations. Age ≥60 years (adjusted hazard ratio (AHR): 1.22; P=0.028), multiple (≥3) procedures during the initial admission (AHR: 2.57; P=0.003), discharge AMA (AHR: 1.68; P=0.047), lack of insurance/self-pay (AHR: 1.23; P=0.034), another coexisting autoimmune disorder (AHR: 1.19; P=0.041), index hospitalizations in the highest income quartile (AHR: 2.05; P=0.006), hyperlipidemia (AHR: 1.89; P=0.026), coexisting kidney disease (AHR: 1.56; P=0.017), and heart failure (AHR: 1.11; P=0.031) were significantly correlated with 30-day readmissions.

Conclusions: SLE lung readmissions were associated with worse outcomes than index hospitalization. Age ≥60 years, multiple procedures, discharge AMA, lack of insurance, kidney disease, and heart failure are significant predictors of readmission.

## Introduction

Systemic lupus erythematosus (SLE) is a chronic immune-mediated disease that predominantly affects women. Approximately 20%-25% of individuals with SLE are hospitalized each year, with hospitalizations primarily due to clinical flares of SLE, infections, thromboembolic disease, or associated medical comorbidities [1]. Early hospital readmissions among SLE patients are common, with SLE ranking as the sixth highest condition for 30-day readmission rates in the United States [1]. The risk of 30-day readmission increases in critical illness and in the presence of comorbidities [2]. The top 50 primary diagnoses associated with 30-day hospital readmissions, categorized by Clinical Classifications Software (CCS) for International Classification of Diseases (ICD), include pneumonia, chronic obstructive pulmonary disease (COPD), bronchiectasis, asthma, pulmonary heart disease, other lower respiratory diseases, respiratory failure/insufficiency/arrest, pleurisy, pneumothorax, and pulmonary collapse [3]. SLE, its organ involvement (including lung involvement), and infections have been identified as common reasons for readmission [4]. Additional significant risk factors for 30-day readmission in the literature include younger age, SLE-related manifestations, and public insurance [5]. Throughout the disease, pulmonary involvement occurs in approximately half of individuals with SLE, presenting with symptoms such as cough and pleural effusion, the latter being the most common radiological abnormality detected [6]. About 63% of SLE patients exhibit respiratory symptoms, and 66% have abnormal lung function [7]. The prevalence of early readmissions following hospitalization for critical illness serves as a metric for evaluating the quality of advanced care and the effectiveness of care transitions. Given the prolonged recovery associated with critical illness and the high risk of severe complications in SLE, it is necessary to explore the factors contributing to readmissions in the immediate post-discharge period. This study examined both all-cause and SLE-specific predictors of 30-day readmissions among critically ill patients hospitalized for lung involvement in SLE.

## Materials and methods

Data source

We analyzed index hospitalizations and 30-day readmissions for lung involvement in SLE from the 2021 Nationwide Readmissions Database (NRD), an extensive repository for hospital readmission data across the United States. The NRD is designed and distributed by the Agency for Healthcare Research and Quality (AHRQ) as part of the Healthcare Cost and Utilization Project (HCUP) to support various types of analyses of national readmissions for all patients, regardless of the expected payer for the hospital stay. This database fills a significant gap in healthcare data by providing nationally representative information on hospital readmissions across all age groups. There are 30 HCUP partner states that contributed to the 2021 NRD. These states are geographically dispersed and account for 61.2% of the total US resident population and 59.6% of all US hospitalizations. The NRD contains data elements that are crucial for generating national estimates of index hospitalizations and readmissions. These include the discharge weights and the discharge strata that are applied to the native data before data analysis. The weighted data thus refers to NRD data that has been expanded to obtain national estimates by the application of discharge weights and strata. The unweighted 2021 NRD includes data on about 16.8 million discharges, while the weighted data approximates 33.4 million discharges. Developed through a federal-state-industry partnership sponsored by the AHRQ, HCUP data inform decision-making at the national, state, and community levels. Beginning in 2016, the National Readmissions Database (NRD) has exclusively adopted the International Classification of Diseases, 10th Revision, Clinical Modification/Procedure Coding System (ICD-10-CM/PCS) for documenting diagnoses and procedures. Each hospitalization in the NRD is recorded as a unique entry with one primary diagnosis (the primary diagnosis for the hospitalization) and up to 35 secondary diagnoses (including comorbidities and acute inpatient complications). The NRD includes over 100 clinical and non-clinical variables for each hospital stay. These include key variables for readmission analysis such as patient linkage numbers for identifying multiple discharges of the same individual, timing between admissions, lengths of hospital stay, and details of transfers and same-day stays. It also includes residency status and variables critical for generating national estimates such as discharge weights and strata for weighting. Diagnoses, procedures, and external causes of morbidity are recorded using ICD-9-CM codes before October 1, 2015, and ICD-10-CM/PCS codes thereafter.

Additional data elements from HCUP software tools, patient demographics, expected payment sources, and financial information such as total charges and hospital costs, which are calculated using the Cost-to-Charge Ratio file, are also part of the database. A detailed description of the NRD and its data elements can be found at https://hcup-us.ahrq.gov/nrdoverview.jsp [8].

The NRD has been shown to provide valuable insights into the influence of demographic factors, such as gender, age, and geography, on readmission prediction in prior research. It helps determine whether there are significant variations in readmission rates among patients with different diseases. Additionally, it explores the characteristics of nationwide hospital admission data and examines how variables such as hospital teaching status, ownership, and location affect readmission rates [9,10].

Data availability

All pertinent data have been included in this manuscript and its supplementary materials. The 2021 NRD data is publicly accessible and can be purchased through the HCUP central distributor upon request at https://hcup-us.ahrq.gov/tech_assist/centdist.jsp or by email request to the AHRQ at hcup@ahrq.gov.

Inclusion and exclusion criteria

The NRD cannot be combined across data years to create a multi-year database as the verified patient linkage numbers (estimated based on a unique combination of the synthetic patient linkage number provided by the HCUP partner, date of birth, and sex) do not track the same person across multiple years. Thus, the NRD documents hospitalizations and readmissions within a single calendar year, without connections to data from previous or subsequent years, enabling the analysis of readmissions exclusively within that specific year. Consequently, we conducted an analysis of adult hospitalizations for lung involvement in systemic lupus erythematosus (SLE) using the 2021 National Readmissions Database (NRD). Critically ill non-elective adult hospitalizations for lung involvement in SLE were identified for analysis using a combination of the International Classification of Diseases, 10th Revision, Diagnostic Code (M32.13 for lung involvement in SLE) and one of any procedure code for mechanical ventilation (5A19XXX), tracheostomy (OB11XXX), extracorporeal membrane oxygenation (5A152XX and 5A15AXX), or bronchoscopy (0BJ08ZZ). Non-lung-related SLE admissions, non-SLE-related lung disorders, patients with concomitant COPD, history of COVID-19 (Z86.16 and U07.1) or severe asthma (ICD-10 code: J45), patients transferred in from other hospitals or admitted for <24 hours, and patients with a do not resuscitate (DNR) order were excluded. Readmissions due to traumatic causes were also excluded [11]. We excluded any planned readmissions and hospitalizations involving coexisting malignancies or immunocompromise, due to their inherently high risk of readmission. Also excluded were discharges with missing or unverified patient linkage numbers; discharges with questionable patient linkage numbers, characterized by having 20 or more discharges in a year, being hospitalized after being discharged as deceased, or having overlapped inpatient stays; and discharges from hospitals where more than 50% of their total discharges were excluded due to any of the aforementioned reasons. Lastly, because NRD data cannot be linked across multiple years, index hospitalizations in December 2021 were excluded as they could not be tracked into the subsequent year. This was done to avoid underestimating the readmission rate by including index admissions in December without subsequent readmissions.

Study end points

The primary end point of this study was the rate and predictors of 30-day readmissions. Secondary end points were the most common reasons for readmission. In addition, we compared the characteristics and resource use between index hospitalizations and 30-day readmissions.

Outcomes and statistical analysis

Statistical analyses were performed using Stata software version 18.0MP (StataCorp LLC, College Station, TX). We conducted our analysis using weighted NRD data to derive nationwide estimates of index hospitalizations and 30-day readmissions for lung involvement in SLE. Specifically, we adjusted for the complex survey design of the NRD by adjusting for clustering, stratification, and weighting using Stata. The NRD's hospital identifier (HOSP_NRD) was specified as the primary sampling unit (PSU) to account for the clustering of patients within hospitals, and the hospital stratum variable (HOSP_STRATUM) was used to adjust for stratification based on geographic region and hospital characteristics. Discharge-level weights (DISCWT) were applied to ensure national representation. The survey design was implemented using the "svyset" command, and all subsequent analyses, including logistic regressions, were conducted with Stata's survey commands (e.g., svy: logit) to obtain appropriate estimates and standard errors that reflect the NRD's sampling structure. This approach was carried out following the guidelines recommended by the HCUP, the NRD's sponsor [12]. We evaluated any differences in baseline demographic characteristics including patient- and hospital-level variables and comorbidities between index hospitalizations and 30-day readmissions using Pearson's chi-squared tests. Using Stata ranking commands, we identified the 20 most common diagnoses associated with readmissions. We estimated the burden of comorbidity using Deyo's modification of the Charlson Comorbidity Index (CCI), which is classified into four levels to signify escalating mortality risk. A CCI score of ≥3 suggests a 10-year mortality risk of 25%, whereas scores of 2 and 1 correlate with substantially lower risks [13]. We estimated the severity of illness using the all-patient refined-diagnosis-related groups (DRG) severity of illness metrics [14]. To identify predictors of readmissions, we used hierarchical Cox proportional hazards regression models. We initially calculated unadjusted hazard ratios from univariable Cox regression analyses, taking into account the different variables and comorbidities as well as predictors of SLE readmissions identified from the literature, such as coexisting autoimmune disease, serositis, chronic kidney disease (CKD), and end-stage renal disease (ESRD), as well as comorbidities such as heart failure and peripheral vascular disease [3,5]. The cutoff for covariate inclusion in subsequent multivariate Cox regression analysis was set at P ≤ 0.2. Predictors of readmissions were identified at P < 0.05 on multivariate Cox regression analysis.

Ethical consideration

The NRD satisfies the highest standards of patient and hospital data confidentiality and privacy. Each hospitalization is de-identified, and the database is publicly accessible. Classified as a limited database with prior ethical approval, this NRD-based study did not require institutional review board approval.

## Results

We analyzed 2,684 index adult hospitalizations for SLE with lung involvement. Among these, 2,641 (98.4%) patients were discharged alive. Of those discharged alive, 593 (22.5%) readmissions were recorded within 30 days. About 137 (23.1%) readmissions were due to lung involvement in SLE, whereas 43 (7.3%) were due to unspecified sepsis. About 40 (6.7%) were due to acute kidney failure, 33 (5.6%) were due to unspecified SLE, 41 (6.9%) were due to hypertension with heart failure with or without CKD, 16 (2.7%) were due to unspecified pneumonia, 12 (2%) were due to fluid overload, and eight (1.3%) were due to pericarditis in SLE. Overall, 189 (31.9%) readmissions were due to complications of SLE. Table 1 summarizes the 20 most recurrent diagnoses associated with 30-day readmissions.

**Table 1:** Top 20 Diagnoses Leading to 30-Day Readmissions Following Hospitalization for Lung Complications in SLE ICD-10: International Classification of Diseases, 10th Revision, N: total readmissions due to specified diagnosis, %: proportion of total readmissions, SLE: systemic lupus erythematosus

Rank	ICD-10 codes	Description	N (%)
1	M32.13	Lung involvement in systemic lupus erythematosus	137 (23.1)
2	A419	Sepsis, unspecified organism	43 (7.3)
3	N179	Acute kidney failure, unspecified	40 (6.7)
4	M329	Systemic lupus erythematosus, unspecified	33 (5.6)
5	I130	Hypertensive heart and chronic kidney disease with heart failure and stage 1 through stage 4 chronic kidney disease, or unspecified chronic kidney disease	18 (3)
6	J189	Pneumonia, unspecified organism	16 (2.7)
7	E8770	Fluid overload, unspecified	12 (2)
8	I120	Hypertensive chronic kidney disease with stage 5 chronic kidney disease or end-stage renal disease	10 (1.7)
9	I110	Hypertensive heart disease with heart failure	8 (1.3)
10	M3212	Pericarditis in systemic lupus erythematosus	8 (1.3)
11	N390	Urinary tract infection, site not specified	7 (1.9)
12	D591	Other autoimmune hemolytic anemias	7 (1.9)
13	M3219	Other organ or system involvement in systemic lupus erythematosus	7 (1.9)
14	K8590	Acute pancreatitis without necrosis or infection, unspecified	6 (1)
15	L03116	Cellulitis of left lower limb	6 (1)
16	N170	Acute kidney failure with tubular necrosis	5 (0.8)
17	I160	Hypertensive urgency	5 (0.8)
18	K254	Chronic or unspecified gastric ulcer with hemorrhage	5 (0.8)
19	R1084	Generalized abdominal pain	5 (0.8)
20	I129	Hypertensive chronic kidney disease with stage 1 through stage 4 chronic kidney disease, or unspecified chronic kidney disease	5 (0.8)

Individuals who were readmitted were younger (median age: 26 versus 31 years; P=0.010) and had a higher proportion of males compared with index hospitalizations (134 (22.6%) versus 459 (17.1%); P=0.007). Readmissions had a greater comorbidity burden compared with index hospitalizations (CCI score ≥ 2: 500 (84.3%) versus 1,938 (72.2%); P<0.001). More readmissions occurred in the third and fourth quarters of the year (316; 53.3%) compared with the first and second quarters for index hospitalizations (1,173 (43.7%); P=0.009). Index hospitalizations had a higher prevalence of hypertension (650 (24.2%) versus 111 (18.7%); P=0.041) and fluid and electrolyte disorders (1,353 (50.4%) versus 260 (43.8%); P=0.031). Readmissions had a higher prevalence of congestive heart failure (146 (24.6%) versus 526 (19.6%); P=0.024), chronic kidney disease (435 (73.3%) versus 1,573 (58.6%); P<0.001), and anemia (136 (23.2%) versus 95 (16.1%); P=0.003) compared with index hospitalizations for lung involvement in SLE. No significant differences were observed in insurance status, baseline illness severity, and mortality risk. Thirty-day readmissions had higher inpatient mortality rates than index hospitalizations (18 (3.1%) versus 43 (1.6%)).

Thirty-day readmissions were associated with longer hospital stays (mean length of stay (LOS): 8 versus 5.8; P<0.001) and higher mean hospital costs (US $84,830 versus US $64,628; P<0.001). Table 2 summarizes other demographic characteristics and resource utilization between index and 30-day readmissions.

**Table 2:** Comparison of Baseline Demographics and Resource Utilization of Index Hospitalizations and 30-day Readmissions Categorical data is presented as frequencies (N) and percentages (%). Continuous data is presented as median with IQR or mean with SD. IQR: interquartile range, SD: standard deviation, LOS: length of hospital stay, THC: total hospital costs, DRG: diagnosis-related groups, AMA: against medical advice, MI: myocardial infarction, PCI: percutaneous coronary intervention, COPD: chronic obstructive pulmonary disease, CABG: coronary artery bypass graft, median household income: median household income for patient's ZIP code, CCI: Charlson Comorbidity Index, CHF: congestive heart failure, CKD: chronic kidney disease, PVD: peripheral vascular disease, LOF: loss of function, DM: diabetes mellitus, DVT: deep vein thrombosis CCI is Sundararajan's adaptation of the modified Deyo's Charlson Comorbidity Index. This adaptation classifies the CCI into four distinct groups, each indicative of escalating comorbidity and mortality risk. A CCI score surpassing 3 is associated with a 10-year mortality rate of approximately 25%, whereas scores of 2 or 1 correspond to 10-year mortality rates of 10% and 4%, respectively. *P<0.05 was considered statistically significant. **Median household income quartiles for patient's ZIP code defined as first quartile = $1-$49,999; second quartile = $50,000-$64,999; third quartile = $65,000-$85,999; and fourth quartile = $86,000 or more.

Variables	Index admissions (N=2,684) (%, unless otherwise specified)	30-day readmissions (N=593) (%, unless otherwise specified)	P*
Median age, year (IQR)	31 (25-42)	26 (23-37)	0.010
Female	2,225 (82.9)	459 (77.4)	0.007
Mean LOS, days	8	5.8	<0.001
Inpatient mortality	43 (1.6)	12 (2)	0.787
Mean THC	US $84,830	US $64,628	<0.001
Insurance status			
Medicare	561 (20.9)	138 (23.2)	0.267
Medicaid	966 (36)	229 (38.6)	0.368
Private	913 (34)	181 (30.5)	0.202
Self-pay	158 (5.9)	32 (5.3)	0.697
CCI			<0.001
1	749 (27.9)	93 (15.7)	-
2	285 (10.6)	57 (9.6)	-
≥ 3	1,653 (61.6)	443 (74.7)	-
DRG severity class**			0.144
Minor LOF	115 (4.3)	14 (2.4)	-
Moderate LOF	488 (18.2)	135 (22.7)	-
Major LOF	1,570 (58.5)	352 (59.3)	-
Extreme LOF	510 (19)	93 (15.7)	-
Admission day is a weekend	561 (20.9)	144 (24.2)	0.192
Median household income (quartile)			
First (0-25th)	950 (35.4)	217 (36.5)	0.679
Second (26th-50th)	714 (26.6)	157 (26.5)	0.959
Third (51st-75th)	644 (24)	151 (25.4)	0.525
Fourth (76th-100th)	346 (12.9)	65 (11.2)	0.316
Metropolitan hospital	2,620 (97.6)	585 (98.6)	0.299
Teaching hospital	2,351 (87.6)	505 (85.2)	0.172
Hospital bed size			0.655
Small	217 (8.1)	52 (8.8)	-
Medium	529 (19.7)	126 (21.3)	-
Large	1,938 (72.2)	415 (69.9)	-
AMA	81 (3)	16 (2.7)	0.745
Discharge quarter			0.009
First	727 (27.1)	122 (20.5)	-
Second	762 (28.4)	156 (26.3)	-
Third	733 (27.3)	184 (31)	-
Fourth	464 (17.3)	132 (22.2)	-
Hospital ownership			0.001
Government	427 (15.9)	56 (9.5)	-
Private, not-for-profit	2,018 (75.2)	467 (78.7)	-
Private, investor-owned	239 (8.9)	70 (11.8)	-
A resident of the same state as the hospital	2,542 (94.7)	581 (97.9)	0.024
Hyperlipidemia	400 (14.9)	80 (13.5)	0.476
Old MI	32 (1.2)	10 (1.7)	0.415
Old PCI	19 (0.7)	8 (1.3)	0.232
Old CABG	11 (0.4)	0 (0)	0.254
Atrial fibrillation	67 (2.5)	17 (2.9)	0.632
Atrial flutter	5 (0.2)	2 (0.3)	0.888
Presence of cardiac pacemaker	5 (0.2)	0 (0)	0.413
COPD	38 (1.4)	14 (2.3)	0.269
Old stroke	118 (4.4)	26 (4.4)	0.977
Acute gout	46 (1.7)	14 (2.3)	0.451
Hypertension	650 (24.2)	111 (18.7)	0.041
DM type 1 and 2	64 (2.4)	11 (1.9)	0.517
PVD	59 (2.2)	14 (2.4)	0.812
Hypothyroidism	59 (2.2)	14 (2.4)	0.812
Oxygen dependence	16 (0.6)	7 (1.1)	0.209
Obesity	204 (7.6)	59 (9.9)	0.178
CHF	526 (19.6)	146 (24.6)	0.024
CKD	1,573 (58.6)	435 (73.3)	<0.001
Chronic liver disease	94 (3.5)	17 (2.9)	0.573
Fluid and electrolyte abnormalities	1,353 (50.4)	260 (43.8)	0.031
Pulmonary circulatory disorders	129 (4.8)	42 (7.1)	0.086
Smoking	11 (0.4)	1 (0.2)	0.473
Anemia	432 (16.1)	138 (23.2)	0.003
Alcohol use disorder	22 (0.8)	4 (0.6)	0.596
Depression	338 (12.6)	80 (13.4)	0.713
DVT	97 (3.6)	22 (3.7)	0.944

Covariates at both patient and hospital levels that showed a significant correlation with readmissions in the univariate Cox regression analysis were included in the multivariate Cox regression model. Factors that correlated with higher likelihood of readmission within 30 days of initial discharge included age ≥ 60 years (adjusted hazard ratio (AHR): 1.22; P=0.028), undergoing three or more procedures during the initial hospital stay (AHR: 2.57; P=0.003), being discharged against medical advice (AMA) (AHR: 1.68; P=0.047), length of index hospital stay ≥ 6 days (AHR: 1.44; P=0.037), and discharges during the second (AHR: 2.55; P=0.016) and third quarters of the year (AHR: 2.25; P=0.043). Additional factors included lack of insurance or self-paying (AHR: 1.23; P=0.034), another coexisting autoimmune disorder (AHR: 1.19; P=0.041), hospitalizations among patients in the highest income quartile (AHR: 2.05; P=0.006), and comorbidities such as hyperlipidemia (AHR: 1.89; P=0.026), chronic kidney disease (AHR: 1.56; P=0.017), and congestive heart failure (AHR: 1.11; P=0.031) (Table 3).

**Table 3:** Uni- and Multivariate Cox Regression Models of Predictors of 30-Day Readmissions Results of univariate and multivariate Cox regression analysis are presented as unadjusted HR and AHR, respectively. The cut-off for inclusion in the multivariate Cox regression was P<0.2 on univariate Cox regression. HR: hazards ratio, AHR: adjusted hazards ratio, HHC: home healthcare, AMA: against medical advice, LOF: loss of function, MI: myocardial infarction, PCI: percutaneous coronary intervention, CABG: coronary artery bypass graft, median household income: median household income for patient's ZIP code, O2: oxygen, SNF: skilled nursing facility, ICF: intermediate care facility, HMO: Health Maintenance Organization, CCI: Charlson comorbidity index, DRG: diagnosis-related groups **Including rheumatoid arthritis, Sjogren's syndrome, antiphospholipid syndrome, autoimmune thyroid disease (Hashimoto's thyroiditis and Graves' disease), myasthenia gravis, autoimmune hemolytic anemia, dermatomyositis or polymyositis, primary biliary cholangitis, celiac disease, and systemic sclerosis

Variables	Univariate Cox regression		Multivariate Cox regression	
	Unadjusted HR	P	Adjusted HR	P
Female	1.15	0.173	0.56	0.081
Age, years				
Age < 18	1.09	0.318	-	-
Age ≥ 18 and < 40	1.83	0.060	1.70	0.436
Age ≥ 40 and < 60	0.61	0.170	1.10	0.867
Age ≥ 60	1.49	0.042	1.22	0.028
Number of procedures				
0-2	0.37	0.005	1.00	-
≥3	2.73	0.005	2.57	0.003
Discharge disposition				
Routine discharge	1.16	0.677	-	-
Discharge to a short-term hospital	1.27	0.751	-	-
Discharge to another facility, SNF, ICF	0.33	0.288	-	-
Discharge to HHC	0.48	0.149	0.47	0.209
Discharged AMA	2.36	0.090	1.68	0.047
Discharge quarter				
First quarter	Reference	Reference	Reference	Reference
Second quarter	2.77	0.008	2.55	0.016
Third quarter	2.26	0.043	2.25	0.043
Fourth quarter	1.88	0.185	2.01	0.145
Hospital control				
Government	Reference	Reference	Reference	Reference
Private, not-for-profit	1.58	0.214	-	-
Private, investor-owned	2.14	0.119	1.47	0.357
Insurance status				
Medicare	Reference	Reference	Reference	Reference
Medicaid	2.56	0.048	2.14	0.133
Private including HMO	2.95	0.021	2.24	0.102
Self-pay	1.53	0.510	1.23	0.034
CCI				
1	Reference	Reference	Reference	Reference
2	0.91	0.832	-	-
≥3	1.21	0.153	1.99	0.033
DRG severity class				
Minor LOF	Reference	Reference	Reference	Reference
Moderate LOF	0.70	0.185	0.80	0.723
Major LOF	0.58	0.093	1.25	0.711
Extreme LOF	0.50	0.227	-	-
Median household income (quartile)				
First (0-25th)	Reference	Reference	Reference	Reference
Second (26th-50th)	1.16	0.667	-	-
Third (51st-75th)	2.37	0.386	-	-
Fourth (76th-100th)	1.44	0.007	2.05	0.006
Metropolitan hospital	2.34	0.402	-	-
Teaching hospital	0.95	0.990	-	-
Admitted on a weekend	0.56	0.104	0.63	0.222
Hospital bed size				
Small	Reference	Reference	-	-
Medium	0.64	0.407	-	-
Large	1.14	0.764	-	-
Hospital ownership				
Government	Reference	Reference	Reference	Reference
Private, not-for-profit	1.58	0.214	-	-
Private, investor-owned	2.14	0.050	1.47	0.357
LOS				
1-2 d	0.78	0.459	-	-
3-5 d	1.60	0.042	0.32	0.296
≥6 d	1.75	0.109	1.44	0.037
Total hospital charge	0.99	0.182	-	-
A resident of the same state as the hospital	2.71	0.169	1.51	0.071
Hyperlipidemia	1.87	0.016	1.89	0.026
Old MI	2.27	0.322	-	-
Old PCI	1.00	-	-	-
Old CABG	1.00	-	-	-
Old pacemaker	1.00	-	-	-
Atrial fibrillation	1.47	0.317	-	-
Atrial flutter	1.00	-	-	-
Old stroke	0.32	0.245	-	-
Hypertension	0.90	0.710	-	-
PVD	1.17	0.571	-	-
Hypothyroidism	1.04	0.916	-	-
DM type 1 and 2	0.38	0.330	-	-
Obesity	1.41	0.440	-	-
CHF	0.48	0.035	1.56	0.017
CKD	1.20	0.431	1.11	0.031
Another autoimmune disorder**	1.39	0.147	1.19	0.041
Liver disease	1.25	0.766	-	-
O_2 _dependence	1.21	0.399	-	-
Fluid and electrolyte disturbance	0.77	0.259	-	-
Pneumonia	2.28	0.160	1.84	0.058
Pericarditis	0.89	0.349	-	-
Smoking	2.65	0.307	-	-
Anemia	1.24	0.495	-	-
Alcohol use disorder	1.00	-	-	-
Depression	1.27	0.247	-	-
Deep venous thrombosis	1.33	0.343	-	-
Pulmonary embolus	0.57	0.143	0.39	0.081

## Discussion

Over the last decade, several studies have examined the rates, predictors, and strategies for reducing 30-day readmissions in patients with SLE. However, to our knowledge, none have focused on critically ill patients requiring intensive care. This study contributes to the existing knowledge in this area by analyzing nationwide hospitalizations of SLE patients requiring intensive care. The study found a high 30-day readmission rate of one in every five SLE patients initially hospitalized to intensive care, rivaling 30-day readmission rates for heart failure (one in every four) [15]. Nearly a quarter of these readmissions were due to exacerbations of pulmonary complications. While other comorbidities also contributed to readmissions, their impact was less pronounced.

The analysis of demographic and clinical characteristics revealed that younger and female patients were more likely to be readmitted; however, after adjusting for covariates, patients aged 60 and older and those with chronic kidney disease and congestive heart failure had a higher likelihood of readmission [16]. The observed gender differences may reflect the higher prevalence of SLE in females, despite males being more prone to severe complications [17].

Prior studies have associated Medicaid insurance and increased damage index to early readmissions among SLE patients [18]. Although these findings were not exactly replicated for those admitted to intensive care, a lack of insurance coverage or self-pay status, along with comorbidities such as congestive heart failure and chronic kidney disease, significantly increased the odds of readmission.

Patients with SLE hospitalized at academic medical centers are generally considered more severely ill than those treated at community hospitals, including large community hospitals in the same region. Conceptually, these patients are thought to be at high risk of adverse outcomes including repeat hospitalizations [19]. While comorbidity index scores of ≥3 were linked to higher odds of readmission, neither admission at metropolitan or academic centers nor illness severity and loss of function during the initial hospitalization showed the same association. This suggests that underlying comorbidities may have a greater impact on readmissions than the quality of critical care.

Sepsis is the leading cause of premature death in SLE patients aged 50 years or younger in the United States, and infection is the most frequent reason for intensive care unit (ICU) admission among SLE patients [20]. As expected, 7% of all readmissions in the study were due to sepsis from an unspecified organism. Sepsis has been reported to account for over 50% of the increase in ICU admissions among SLE hospitalizations over the past two decades [21,22]. The role of sepsis in readmissions in this cohort may be linked to the risk of post-sepsis syndrome, which is more common among individuals who have been admitted to an ICU or hospitalized for extended periods. Infections can mimic SLE illness flare-ups, making diagnosis and therapy difficult. Several techniques have been explored to reduce infection risk in SLE patients, including vaccination, antibiotic/antiviral prophylaxis, and intravenous immunoglobulin therapy. The European Alliance of Associations for Rheumatology (EULAR) guidelines recommend immunizations for the prevention of infections (herpes zoster virus, human papillomavirus, influenza, COVID-19, and pneumococcus), management of bone health, nephroprotection and cardiovascular risk, and screening for malignancies in patients with SLE [23]. However, in practice, managing SLE and preventing infections presents a significant challenge, as many of the treatments necessary for controlling severe or refractory disease, such as intravenous cyclophosphamide and rituximab, inherently increase the risk of infections. While these therapies are critical for managing organ-threatening conditions, their immunosuppressive effects make patients more susceptible to infections and sepsis. Achieving disease control while managing infection risk is crucial given the nature of SLE and the limitations of current treatments. Sepsis is expected to remain a significant comorbidity in SLE readmissions, making its prevention particularly important as the American College of Rheumatology (ACR) prepares to release new recommendations in the spring of 2025.

Patients with SLE often present with additional autoimmune disorders, with research indicating that up to 45% of these patients are affected by at least one other autoimmune condition [24]. These coexisting autoimmune disorders, such as rheumatoid arthritis and antiphospholipid syndrome, significantly contribute to the increased readmission rates observed in SLE patients by intensifying the complexity and burden of their disease. The presence of these additional conditions often results in more severe clinical manifestations, greater treatment challenges, and an increased susceptibility to complications. This complex interplay of autoimmune diseases often necessitates more intensive management strategies and is a key factor in the elevated risk of recurrent hospitalizations. For critically ill SLE patients, the risk of rehospitalization is further compounded by comorbidities, complications encountered during the initial hospitalization (such as delirium and extended mechanical ventilation), and the occurrence of subsequent infections after discharge. Addressing pre-existing comorbidities and ensuring careful management during care transitions are crucial to reducing healthcare utilization post-critical care discharge. Future research should focus on developing targeted interventions for at-risk critical care survivors with SLE to mitigate the risk of subsequent rehospitalization.

Table 4 summarizes the studies that have examined the rates, predictors, and strategies for reducing 30-day readmissions in patients with SLE.

**Table 4:** Related Evidence on SLE Readmission Over the Last Decade (Select) RCT: randomized controlled trial, SLE: systemic lupus erythematosus, HF: heart failure, ESRD: end-stage renal disease, SLEDAI: Systemic Lupus Erythematosus Disease Activity Index, ED: emergency department

S/N	Title	Takeaway	Authors	Year	Study type	Journal	DOI
1	Thirty‐day hospital readmissions in systemic lupus erythematosus: predictors and hospital‐ and state‐level variation	Thirty-day hospital readmissions in SLE are influenced by patient, hospital, and geographic factors, with higher readmission rates in rural areas.	Yazdany et al. [3]	2014	Non-RCT observational study	Arthritis & Rheumatology	10.1002/art.38768
2	Effects of transitional care on self-care, readmission rates, and quality of life in adult patients with systemic lupus erythematosus: a randomized controlled trial	Transitional care improves self-care and quality of life in adult patients with SLE and reduces readmissions compared to usual care	Xie et al. [25]	2018	RCT	Arthritis Research and Therapy	10.1186/s13075-018-1670-4
3	Causes and predictors of early hospital readmission in systemic lupus erythematosus	Early hospital readmission in SLE patients is more likely to occur due to government-sponsored Medicaid insurance, increased damage index, and lower serum hemoglobin and albumin levels.	Nangit et al. [1]	2018	Non-RCT observational study	The Journal of Rheumatology	10.3899/jrheum.170176
4	The relationship between remission and health-related quality of life in a cohort of SLE patients	Remission in SLE patients is strongly associated with improved physical quality of life, but not mental quality of life.	Tsang-A-Sjoe et al. [26]	2018	Non-RCT observational study	Rheumatology	10.1093/rheumatology/key349
5	Quality improvement intervention to reduce thirty‐day hospital readmission rates among patients with systemic lupus erythematosus	A multidisciplinary post-discharge intervention can effectively reduce 30-day readmission rates among patients with systemic lupus erythematosus.	Bowers et al. [27]	2020	Quality improvement initiative	Arthritis Care & Research	10.1002/acr.24435
6	One-quarter of Medicare hospitalizations in patients with systemic lupus erythematosus readmitted within thirty days	Thirty-day readmissions in patients with SLE are comparable to HF, suggesting that readmission prevention programs should focus on young ESRD patients with SLE.	Bartels et al. [2]	2021	Non-RCT observational study	Seminars in Arthritis and Rheumatism	10.1016/j.semarthrit.2021.02.006
7	Risk factors for one-year hospital readmissions in patients with systemic lupus erythematosus	SLE patients with older age of onset, low serum albumin levels, and high cystatin C levels are at higher risk for readmission due to infections and lupus activity.	Chen et al. [28]	2021	Single-center, retrospective case-control study	Research Square	10.21203/rs.3.rs-989689/v1
8	2021 DORIS definition of remission in SLE: final recommendations from an international task force	The DORIS Task Force recommended a single definition of remission in SLE, based on clinical SLEDAI=0, Evaluator’s Global Assessment <0.5 (0-3), prednisolone 5 mg/day or less, and stable antimalarials, immunosuppressives, and biologics.	van Vollenhoven et al. [29]	2021	Systematic review	Lupus Science & Medicine	10.1136/lupus-2021-000538
10	Prevalence of hospital readmissions and related factors in patients with autoimmune diseases	Autoimmune patients are more likely to be readmitted to the hospital, with arterial hypertension and previous use of immunosuppressants being associated factors.	Morales-Tisnés et al. [30]	2021	Retrospective cohort study	Journal of Translational Autoimmunity	10.1016/j.jtauto.2021.100121
12	Predictors of thirty-day hospital readmissions in systemic lupus erythematosus in the united states: a nationwide study	The strongest risk factors for 30-day readmission were younger age, SLE-related manifestations, and public insurance.	Najjar et al. [5]	2022	Retrospective cohort study	Arthritis Care and Research	10.1002/acr.24900
13	Health care utilization in systemic lupus erythematosus in the community: the Lupus Midwest Network	Patients with SLE have significantly higher rates of hospitalization and ED visits compared to individuals without SLE, across all sexes and age groups.	Chevet et al. [31]	2022	Population-based cohort study	Journal of Clinical Rheumatology	10.1097/RHU.0000000000001899
14	Age-stratified 30-day rehospitalization and mortality and predictors of rehospitalization among patients with systemic lupus erythematosus: a Medicare cohort study	Young adults with SLE on Medicare have exceptionally high 30-day rehospitalization rates, at 36%, which is significantly higher than their peers without SLE and older SLE patients.	Schletzbaum et al. [32]	2023	-	Journal of Rheumatology	10.3899/jrheum.220025
15	Association between frailty status and readmissions in hospitalized patients with systemic lupus erythematosus	Among hospitalized adults with SLE, the presence of frailty was associated with higher readmission and inpatient mortality rates.	Leung et al. [33]	2024	Retrospective cohort study	ACR Open Rheumatology	10.1002/acr2.11722
16	Outcomes of patients newly-diagnosed with systemic lupus erythematosus managed in a tertiary training and referral hospital in the Philippines	Newly diagnosed SLE patients in Manila revealed low remission rates and significant organ damage within the first year. Most hospitalizations and all deaths occurred during the initial hospitalization.	Suilan et al. [34]	2024	Retrospective cohort study	Acta Medica Philippina	10.47895/amp.vi0.5896

Limitations and strengths

The National Readmissions Database captures hospitalization and readmissions data for a single calendar year, which prevents the tracking of individual patients over multiple years. This limitation restricts the ability to monitor long-term outcomes for critical care survivors with SLE using this database. Consequently, hospitalizations in December 2021 were excluded since they could not be tracked into 2022. In addition, the database does not include crucial information such as the duration of critical care admissions, medication history, or laboratory results, which hinders a comprehensive assessment of how these factors may influence rehospitalization. However, the index study draws from a large, hospital-based, multi-ethnic registry, providing a diverse and representative SLE sample. Additionally, the study examines multiple hospital- and patient-level covariates related to SLE admissions and readmissions, offering a comprehensive and in-depth analysis of the burden that SLE and its associated readmissions place on the US healthcare system, underscoring the seriousness of SLE-related readmissions.

## Conclusions

The burden of unplanned 30-day readmissions among critical care survivors hospitalized for lung involvement in SLE is substantial with one in five readmitted within 30 days. Our findings indicate that readmissions are associated with poorer outcomes, including higher inpatient mortality, longer hospital stays, and increased healthcare costs. The most common reasons for readmission were recurrent lung involvement and other SLE-related complications. Key predictors of readmission include advanced age, the need for multiple procedures during the initial hospitalization, discharge against medical advice, lack of insurance, and coexisting kidney disease or heart failure. There is a need for the development of tailored interventions to reduce readmission rates and improve outcomes for this subset of SLE patients.
